# Spontaneous nosocomial *Proteus mirabilis* meningitis in a Human Immunodeficiency Virus (HIV)-infected adult patient: a case report

**DOI:** 10.1186/s13256-022-03704-0

**Published:** 2023-02-09

**Authors:** Radhika Sood, Chadrack Walo, Rosie Burton, Mohamad Khalife, Astan Dicko, Freddy Mangana

**Affiliations:** 1 Internal Medicine Department, Hôpitaux Universitaires de Genève, Geneva, Switzerland; 2 Centre Hospitalier Kabinda, Médecins Sans Frontieres, Commune de Lingwala, Kinshasa, Democratic Republic of Congo; 3 SAMU, Médecins Sans Frontieres, Kinshasa, Democratic Republic of Congo; 4 Médecins Sans Frontieres, Kinshasa, Democratic Republic of Congo; 5 Laboratory of Centre Hospitalier Kabinda, Médecins Sans Frontieres, Kinshasa, Democratic Republic of Congo

**Keywords:** *Proteus mirabilis*, Gram-negative bacillary meningitis, Advanced HIV, Healthcare-associated meningitis

## Abstract

**Background:**

Gram-negative bacillary meningitis remains a rare occurrence, even in patients with human immunodeficiency virus. Current literature only describes anecdotal cases of spontaneous nosocomial *Proteus mirabilis* meningitis. This report describes the clinical manifestations and management of a patient with healthcare-associated spontaneous Gram-negative bacillary meningitis in a patient with advanced human immunodeficiency virus disease.

**Case presentation:**

A 23-year-old Congolese female was hospitalized in a human immunodeficiency virus specialized center for ongoing weight loss, chronic abdominal pain, and vomiting 9 months after initiation of treatment for tuberculosis meningitis. Hospitalization was complicated by healthcare-associated Gram-negative bacillary meningitis on day 18. Blood and cerebrospinal fluid cultures confirmed *Proteus mirabilis*. Antibiotic susceptibility testing showed extended spectrum beta-lactamase resistant to common antibiotics, and sensitive to meropenem. Despite initiation of high-dose meropenem by intravenous infusion (2 g every 8 hours), the patient did not improve, and died after 4 days of meropenem treatment. Gram-negative bacillary meningitis remains rare and is associated with an unfavorable prognosis.

**Conclusions:**

This case report highlights the importance of microbiological identification of pathogens in resource-limited settings. As Gram-negative bacillary meningitis does not present with pleocytosis in patients with advanced human immunodeficiency virus, a negative lumbar puncture cannot exclude this diagnosis. Access to clinical bacteriology in resource-limited settings is essential to enable correct antibiotic treatment and avoid overuse of antibiotics to which there is already resistance. It further plays an essential role in public health by identifying antibiotic susceptibilities. Infection prevention and control measures must be reinforced in order to protect patients from such avoidable healthcare-associated infections.

## Background

Gram-negative bacillary meningitis remains a rare occurrence, even in patients with advanced human immunodeficiency virus (HIV) disease (AHD) [1]. This report describes the clinical manifestations and management of healthcare-associated spontaneous Gram-negative bacillary meningitis in a patient with AHD. *Proteus mirabilis* is a Gram-negative bacillus, known for its characteristic motility and urease production [2]. Frequently associated with urinary tract infections and catheter biofilm formation, it is rarely incriminated in meningitis. We report a case of spontaneous healthcare-associated *Proteus mirabilis* meningitis in an immunocompromised individual.

## Case presentation

**Fig. 1:**
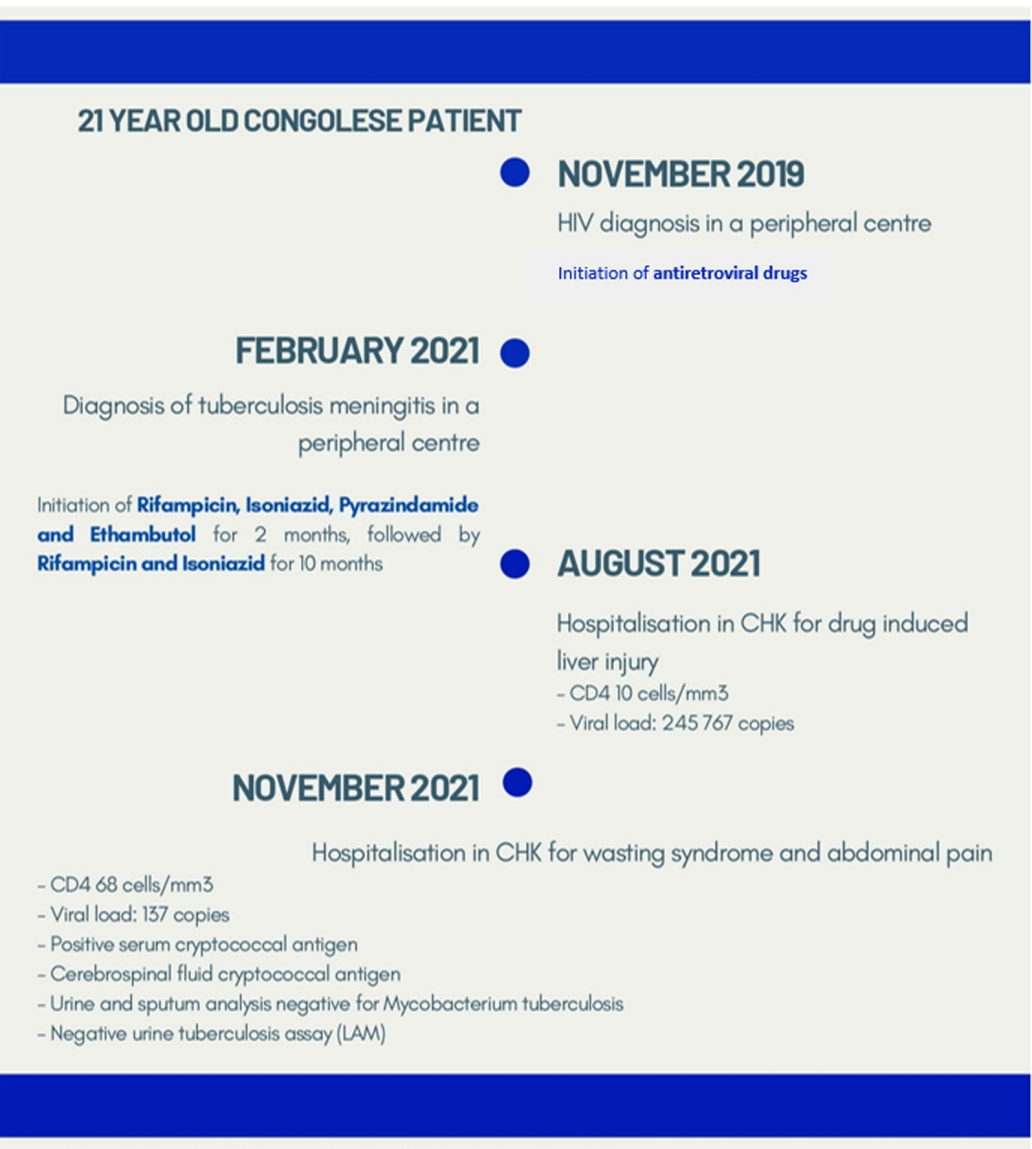
Patient timeline prior to hospitalization in November 2021

This case report presents a 23-year-old Congolese female patient with HIV on first line antiretroviral drugs (tenofovir–lamivudine–dolutegravir) since November 2019 following HIV diagnosis in a tertiary center. Underlying conditions included tuberculosis (TB) meningitis, which was under treatment since February 2021. The patient trajectory is outlined in Fig. 1.

The patient history revealed that initial symptoms were dominated by headaches. The initial clinical presentation and the investigations relevant to the initial diagnosis, as well as cluster of differentiation 4 (CD4) cells and viral load at treatment initiation are all unknown. The TB meningitis diagnosis and treatment were initiated at another center as per Congolese guidelines. National guidelines for tuberculosis meningitis recommend treatment for 12 months with an intensive phase of quadruple therapy (rifampicin, isoniazid, pyrazinamide, and ethambutol) for 2 months, followed by rifampicin and isoniazid for 10 months [3]. Neurological symptoms improved soon after the initiation of treatment.

The patient was hospitalized in August 2021 in Centre Hospitalier Kabinda (CHK). CHK is a hospital based in Kinshasa, Democratic Republic of Congo, which is run by Médecins sans Frontières (MSF). Hospitalization was motivated by drug-induced liver injury, likely due to TB medication (while she was on continuation phase treatment of rifampicin and isoniazid). The differential diagnosis of drug-induced liver injury secondary to cotrimoxazole was also suggested, which she was taking for prophylaxis of opportunistic infections. At the time of admission, CD4 levels were at 10 cells/mm^3^ with a 6-digit viral load (245,767 copies/ml), and administration of rifampicin, isoniazid, and cotrimoxazole was discontinued. Following improvement, the TB medications were successfully rechallenged and the patient was discharged. As per local protocols, cotrimoxazole was not rechallenged.

During this hospitalization at CHK, the patient received a psychosocial evaluation by specialized nurses, which revealed poor compliance to HIV and TB treatment.

The patient was rehospitalized in CHK in November 2021. This hospitalization was motivated by severe abdominal pain and wasting syndrome. Routine testing revealed positive serum cryptococcal antigen status, with CD4 levels at 68 cells/mm^3^and a viral load of 137 copies/ml. The patient had no neurological symptoms and cerebrospinal fluid cryptococcal antigen was negative. Further investigations found no evidence of drug-resistant TB, with samples of urine and sputum negative for *Mycobacterium tuberculosis* resistance to rifampicin (MTB-RIF). Urine *Mycobacterium tuberculosis* cell wall antigen lipoarabinomannan (TB-LAM) on admission was negative.

Due to severe nausea and vomiting, all oral treatments were interrupted upon hospitalization in spite of the lack of clinical parameters in favor of recurring drug-induced liver disease. Only antiemetic drugs were given. She had clinical evidence of malnutrition with a rash typical of pellagra, which responded to treatment with niacin. Serum protein and albumin levels could not be measured, as these parameters were not measured in the local laboratory. The patient’s trajectory was initially favorable, as the nausea and vomiting subsided and her appetite improved. The cause of the initial degradation was not found.

Hospitalization was complicated on day 18 by 39 °C fever with altered level of consciousness. Clinical examination revealed a Glasgow scale of 9/15 (eye response 3 points, verbal response 1 point, motor response 5 points) with no focal abnormality of the cranial nerves or long tracts. There was no nuchal rigidity. The patient presented with polyserositis with multiple third space fluid collections (ascites, pleurisy, and generalized edema), none of which were accessible to diagnostic tap. Skin examination revealed no open wounds or signs of infected intravenous catheters. Due to concern about nosocomial bacterial infection, empiric antibiotics (amikacin, azithromycin, and vancomycin) were started after samples were obtained for cerebrospinal fluid (CSF) and blood cultures (two samples routinely taken for blood culture according to local protocols).

### Investigations

Upon deterioration on day 18 of hospitalization, blood investigations showed stable inflammatory normochromic normocytic anemia at 8.6 g/dl and hyperleukocytosis at 15.7 × 10^9^/L (88% neutrophilic). Liver enzymes revealed mild cytolysis with persistent but improving cholestasis, as shown in Table 1.

**Table 1:** Laboratory parameters

Laboratory parameter	Normal range	On admission (day 0)	On deterioration (day 18)
Serum glutamic pyruvic transaminase	7–56 mmol/l	46.2	52.3
Total bilirubin	0.1–1.2 mg/dl	5.5	2.9
Direct bilirubin	< 0.3 mg/dl	5.2	2.8
Gamma glutamyl transferase	5–40 UI/L	137	129
Serum alkaline phosphatase	30–120 UI/L	85.2	75.3
Serum creatinine	53–97.2 mcmol/l	19.4	52.3
Serum sodium	135–145 mmol/l	123	119

Other results showed acute renal failure, hypoosmolar hyponatremia, and normal potassium levels. Lumbar puncture revealed normal opening pressure, three white blood cells that were not further identified, low glucose (normal range 35–57.5 mg/dl), a negative Pandy’s test showing that protein was not above the normal range (positive Pandy’s test, total cerebrospinal fluid protein > 0.45 g/litre) and negative cryptococcal CSF antigen. Further imaging was not possible due to the lack of local portable resources and inability to transport the patient, who was in a critical state.

**Fig. 2:**
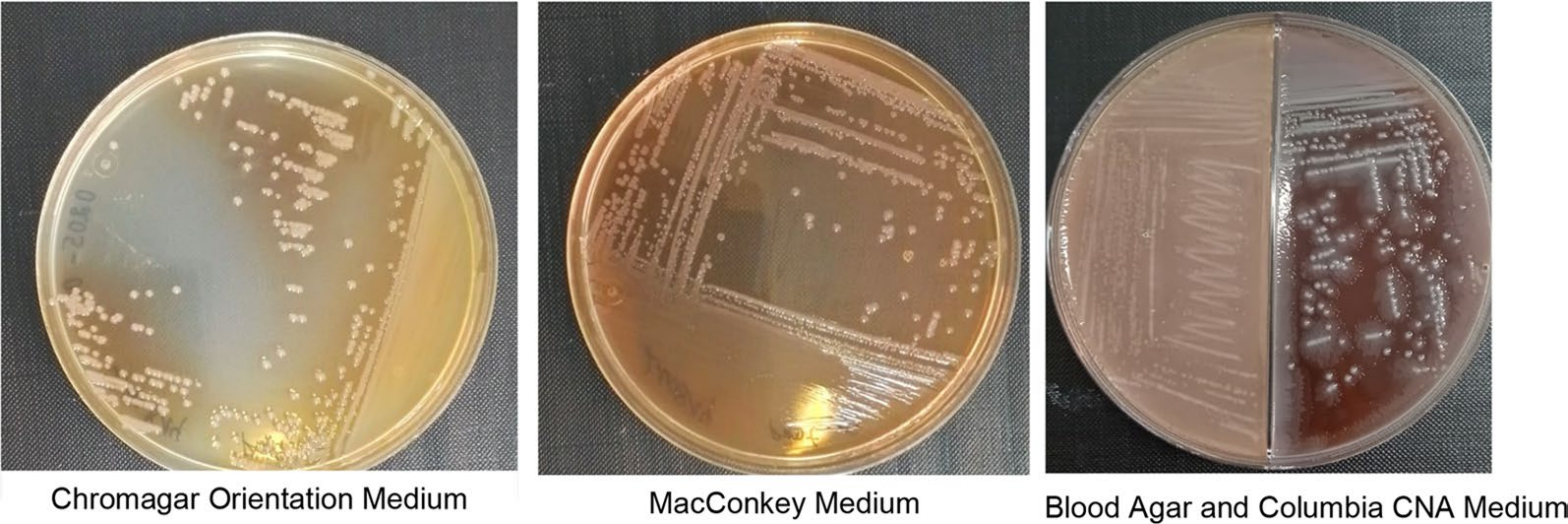
*Proteus mirabilis* colonies on Petri dishes (culture medium as indicated)

**Fig. 3:**
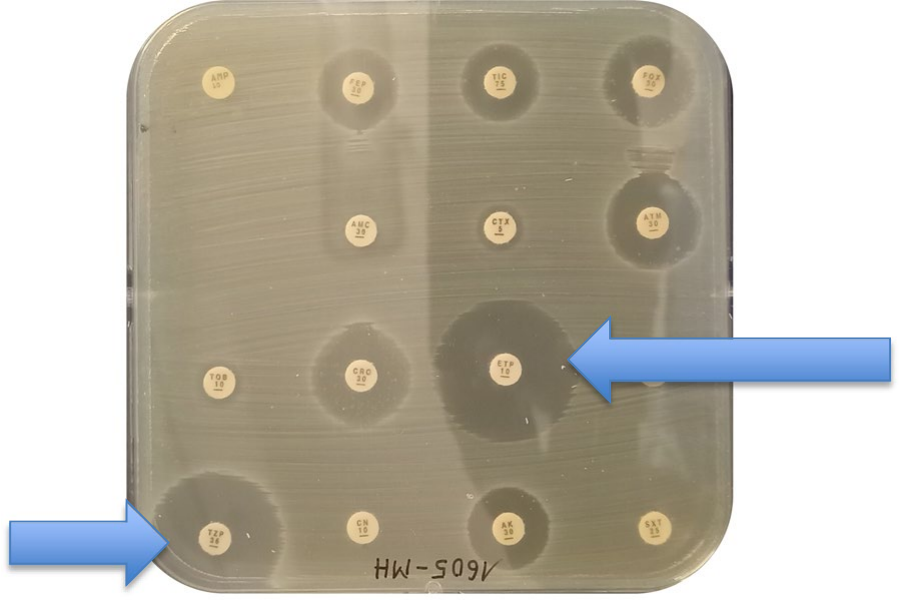
Antibiotic susceptibility testing: Petri dish with Mueller–Hinton medium. Blue arrows indicate antibiotic susceptibility. *ETP* Ertapenem, *TZP* Piperacilline-Tazobactam

Blood and cerebrospinal fluid cultures revealed Gram-negative bacilli within 24 hours of sampling on the Gram stain. Further characterization confirmed *Proteus mirabilis* in blood and CSF cultures (Fig. 2). The strain was shown to be sensitive only to piperacilline/tazobactam and meropenem (Fig. 3). Piperacilline/tazobactam does not penetrate the blood–brain barrier and so was not a treatment option [4].

Once a culture vial is detected positive, different mediums can be used in order to further characterize the pathogen. Figure 2 shows four different culture mediums with *Proteus mirabilis* colonies from the presented patient. The CHROmagar Orientation medium is a non-selective agar. The MacConkey medium is a selective medium for Gram-negative bacteria that further characterizes pathogens on the basis of lactose fermentation. The majority of *Enterobactericae* ferment lactose, with some exceptions that include *Proteus mirabilis*. The cerebrospinal fluid was cultivated on the blood agar and Colistin Nalidixic Acid (CNA) medium. The left half of the Petri dish contains Columbia on medium boiled blood agar, while the right half contains Columbia CNA blood agar. The antibiotics inhibit the growth of Gram-negative bacilli. Figure 2 shows resistance to Nalidixic acid, as confirmed in Fig. 3 and Table 2 (resistance to ciprofloxacin, which is of the same family).

**Table 2:** Interpretation of antibiotic susceptibilities (antibiotics indicated in the same order as Fig. 3)

*AMP 10 µg* *Ampicilline*	*FEP 30 µg* *Cefepime*	*TIC 75 µg* *Ticarcillin*	*FOX 30 µg* *Cefoxitin*
	*AUG 20 + 10 µg* *Amoxicilline + clavulanate*	*CTX 5 µg* *Cefotaxime*	*ATM 30 µg* *Aztreonam*
*TM 10 µg* *Tobramycin*	*CRO 30 µg* *Ceftriaxone*	**ETP 10 µg** **Ertapenem**	*CIP 5 µg* *Ciprofloxacin*
**TZP 30 + 6 µg** **Piperacilline–tazobactam**	*CN 10 µg* *Gentamycin*	*AK 30 µg* *Amikacin*	*SXT 1.25 + 23.75 µg* *Cotrimoxazole*

In Italic: resistant to the indicated antibiotic

In bold: sensitive to the indicated antibiotic

AMP = Ampicilline, FEP = Cefepime, TIC = Ticarcillin, FOX = Cefoxitin, AUG = Amoxicilline + clavulanate, CTX = Cefotaxime, ATM = Aztreonam, TM = Tobramycin, CRO = Ceftriaxone, ETP = Ertapenem, CIP = Ciprofloxacin, TZP = Piperacilline–tazobactam, CN = Gentamycin, AK = Amikacin, SXT = Cotrimoxazole

Suspension of the colonies extracted from Fig. 2, then spread on a Petri dish on a Mueller–Hinton medium, as shown in Fig. 3. Antibiotic disks of different concentrations were then placed in the box to determine antibiotic susceptibility.

**Fig. 4:**
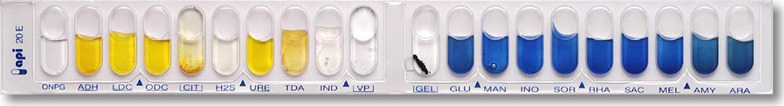
Analytical profile index (API20E)

**Fig. 5:**
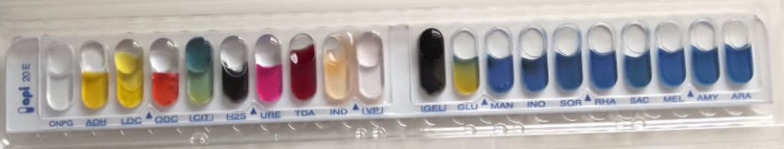
API20E (analytical profile index 20E) online database

Figure 4 shows the analytical profile index (API) 20E, a standardized test that contains 20 biochemical tests [5]. It is used to identify Gram-negative bacilli that belong to the *Enterobacteriacae* family, as well as other non-fastidious Gram-negative bacteria. Each chamber contains a dehydrated substrate, which is rehydrated by the bacterial suspension, allowing for incubation. Resulting color changes are compiled to obtain a profile number that is compared with an online repository to identify the bacterial species (Fig. 5).

### Differential diagnosis

Initially, many differential diagnoses were considered in light of the acute febrile encephalopathy presented in a nosocomial setting.

Among infectious causes, recurring TB meningitis was considered. We evaluated this diagnosis to be unlikely given she had received 9 months of treatment for TB, and that her neurological symptoms had resolved soon after starting treatment. Toxoplasmosis or cryptococcal meningitis also appeared less likely due to the absence of focal neurological signs and negative cryptococcal antigen in the cerebrospinal fluid. Rapid plasma reagin analysis and the rapid diagnostic test for malaria was negative, rendering neurosyphilis and cerebral malaria less likely. Viral encephalitis remained a potential differential diagnosis, but polymerase chain reaction (PCR) confirmation was not available.

Neurological tuberculosis-immune reconstitution inflammatory syndrome (TB-IRIS) was not considered as a differential diagnosis due to the absence of reintroduction of antiretrovirals. The patient was on antiretrovirals since November 2019, but all oral treatments were suspended upon admission due to poor oral tolerance. No medications or metabolic causes were incriminated (normal glycemic control, as well as liver and kidney function).

Septic encephalopathy remained the working diagnosis and culture results within 48 hours diagnosed nosocomial *Proteus mirabilis* meningitis. Blood (two aerobic cultures) and cerebrospinal fluid cultures detected the same serotype of the pathogen.

The source of infection was not identified. Prior to clinical deterioration, the patient did not have a urinary catheter or complaint of upper or lower urinary tract symptoms. No prior neurosurgical procedure had been performed. Throughout the hospitalization, the patient was equipped with a peripherally inserted venous catheter which was changed regularly as per the local protocol.

### Treatment

Following initial clinical deterioration, the patient was transferred to the intensive care unit for monitoring with initiation of infection prevention and control precautions for multidrug-resistant bacteria. Empiric antibiotics (amikacin, azithromycin, and vancomycin) were started according to local antibiotic stewardship protocols and availability. While we judged a bacterial infection likely, meningitis was considered unlikely. Due to limited availability of meropenem, local protocols restricted its use to culture results, showing sensitivity to meropenem with resistance to other available antibiotics. In our patient, following culture results, intravenous meropenem 2 g (administered over 30 minutes) every 8 hours was introduced (extended infusion was not applied due to nursing constraints). In spite of antibiotic treatment, the patient developed hemodynamic instability requiring vasoactive amine use (noradrenaline), which was initiated by a peripheral intravenous catheter and titrated to allow for a mean arterial pressure of 60 mmHg.

### Outcome and follow-up

The patient did not improve, Glasgow scale deteriorated to 6/15. The patient died on day 4 following initiation of meropenem. Written informed consent was obtained *post mortem* from the patient’s sister.

## Discussion and conclusions

CHK is a MSF center providing free healthcare for patients with HIV in Kinshasa, Democratic Republic of Congo. It is a 60-bed facility with an intensive care capacity of 6 beds. The patient cohort is made up of patient with advanced HIV. The hospital has been equipped with a microbiology laboratory since August 2021. Previously, all samples were referred to a local private laboratory. Widespread bacterial resistance is extensive, for both community and nosocomial infections. Manual methods are used for bacterial identification and sensitivity determination. According to existing protocols, routine blood culture is performed on admission if clinical presentation includes fever or shock, or if bacterial infection is suspected. Blood cultures are performed during hospitalization in case of suspicion of a nosocomial infection. Since November 2021, routine cerebrospinal fluid culture is performed.

Meningitis remains common in patients with HIV/AIDS. The risk of developing spontaneous bacterial meningitis in HIV-infected individuals remains 19 times higher than in the general population [6]. While any pathogen can be encountered, patients with advanced HIV are often infected by *Cryptococcus neoformans*, *Mycobacterium tuberculosis*, and bacterial meningitis. Common causes of community-acquired bacterial meningitis are similar in patients with HIV when compared with the general population, that is, *Streptococcus pneumonaie* and *Neisseria meningitis* [7].

Gram-negative bacillary meningitis has been described in three different forms: pediatric [8–10], secondary to trauma or neurosurgery [11], or spontaneous. No specific association to HIV has been described in existing literature. This case report focuses on spontaneous nosocomial Gram-negative bacillary meningitis in a patient with advanced HIV. It is the first case of *Proteus mirabilis*-associated meningitis described in CHK.

A South African retrospective cohort study of 26 adult patients hospitalized with spontaneous Gram-negative bacillary meningitis over 2 years described one case of nosocomial acquired *Proteus mirabilis* meningitis in a patient infected with advanced HIV. This study interestingly underlined the lack of cerebrospinal spinal fluid pleocytosis in patients with advanced HIV presenting with Gram-negative bacillary meningitis [1], as encountered in the presented case report. A possible delay in neutrophilic increase was postulated as a theory to explain the latter, but not confirmed by serial spinal taps. This highlights a key point—that normal cerebrospinal fluid analysis cannot exclude bacterial meningitis.

*Proteus mirabilis* is a Gram-negative rod-shaped bacterium belonging to the *Enterobacteriaceae* family [2]. While the source of infection was not identified in our patient, *Proteus mirabilis* can be found in multiple environments, including soil and water sources. It is a commensal organism of human and animal gastrointestinal tracts. It is identified by the characteristic bull’s eye pattern of motility on agar plates. The urease activity results in alkalinizing properties responsible for frequent urolithiasis, which are a continuous source of bacteria.

A retrospective review of *P. mirabilis* infections in hospitalized patients over 2 years found that infections were nosocomial in 61% of cases [12]. The patient population, however, did not include patients with advanced HIV. Bacteria was isolated predominantly from urine samples (70%). *Proteus mirabilis* is typically encountered in catheter-associated urinary tract infections, which are often polymicrobial. This is likely due to its biofilm formation properties. Other sources included wounds (12%), bronchopulmonary samples (6%), and blood cultures (2%) [12].

Gram negative bacillary meningitis remains rare even in patients with advanced HIV. The most common pathogens are *Escherichia coli* and *Pseudomonas* species. In patients with advanced HIV, *Klebsiella pneumonaie* and non-typhoidal *Salmonella* meningitis have also been described [1, 13]. A large prospective trial over a 25-year period of Gram-negative bacillary meningitis found that spontaneous cases mainly occur in patients with underlying conditions, including alcoholism, cirrhosis, diabetes, and immunosuppression [13]. Prognosis is typically unfavorable with high morbidity (severe neurological handicap) and mortality.

While community-acquired meningitis typically results from *Streptococcus pneumoniae* and *Neisseria meningitis*, the distribution of healthcare-associated pathogens is different. A retrospective review over 14 years of 326 nosocomial infections found 52% of Gram-positive organisms and 48% of Gram-negative [14]. The majority of patients had undergone recent neurosurgery.

A retrospective analysis of 10 French intensive care units over 16 years published in 2006 included 40 adults admitted for spontaneous Gram-negative bacillary meningitis, excluding *Haemophilius influenzae*. Spontaneous *Proteus mirabilis* infection was found in two cases [15]. This study suggested that intestinal parasitosis (including strongyloidiasis) was a predisposing factor.

Gram-negative bacillary meningitis is a rare occurrence in adults; current literature only revealed anecdotal cases of spontaneous nosocomial *P. mirabilis* meningitis. This case highlights the utility of microbiological diagnosis in resource-limited settings. Gram-negative bacillary meningitis does not present with pleocytosis in patients with advanced HIV, and a negative lumbar puncture cannot exclude this diagnosis. Access to clinical bacteriology in resource-limited settings is essential to enable correct antibiotic treatment and avoid overuse of antibiotics to which there is already resistance. It further plays an essential role in public health by identifying antibiotic susceptibilities. Infection prevention and control measures must be reinforced in order to protect patients from such avoidable hospital acquired infections.

## Learning points/take-home messages

- Gram-negative bacillary meningitis remains rare
- This is one of four known cases of spontaneous, healthcare-associated *Proteus mirabilis* meningitis found in literature, and the second such case in a patient with advanced HIV
- *Proteus mirabilis* meningitis is associated with poor prognosis
- Gram-negative bacillary meningitis may not present with pleocytosis in patients with advanced HIV, and a negative lumbar puncture cannot exclude this diagnosis
- Consider initiation of carbapenems for severely immunocompromised patients with severe sepsis due to nosocomial infections
- Infection prevention and control measures must be reinforced to protect patients from avoidable hospital acquired infections
- This report shows the need for improving access to microbiological diagnosis in resource limited settings in the context of increasing antibiotic resistance

## Data Availability

Not applicable.

## Abbreviations

AHD: Advanced human immunodeficiency virus disease
CHK: Centre Hospitalier Kabinda
*P. mirabilis*: *Proteus mirabilis*
HIV–AIDS: Human immunodeficiency virus–Acquired immunodeficiency syndrome
MSF: Médecins sans Frontières (Doctors Without Borders)
MTB–RIF: Mycobacterium–rifampicine
TB-LAM: Tuberculosis lipoarabinomannan
CSF: Cerebrospinal fluid
TB-IRIS: Tuberculosis-immune reconstitution inflammatory syndrome
PCR: Polymerase chain reaction
